# A phase I dose-escalation study to evaluate safety and tolerability of sorafenib combined with sirolimus in patients with advanced solid cancer

**DOI:** 10.1038/sj.bjc.6605777

**Published:** 2010-11-02

**Authors:** I M E Desar, J N H Timmer-Bonte, D M Burger, W T A van der Graaf, C M L van Herpen

**Affiliations:** 1 Department of Medical Oncology 452, Radboud University Nijmegen Medical Centre, PO Box 9101, Nijmegen, 6500 HB, The Netherlands; 2 Department of Clinical Pharmacy, Radboud University Nijmegen Medical Centre, Nijmegen, The Netherlands

**Keywords:** phase I, sorafenib, sirolimus, tyrosine kinase inhibitor, mTOR inhibitor

## Abstract

**Background:**

The combination of sorafenib (vascular endothelial growth factor receptor 2 inhibitor) and sirolimus (mammalian target of rapamycin inhibitor) might work synergistically.

**Methods:**

A phase I dose-escalation study with sorafenib twice a day (b.i.d.) and sirolimus once daily (q.d.) was performed to determine the recommended dose of the combination in patients with solid tumours. Secondary objectives were to determine the safety profile and maximum tolerated dose (MTD), and to evaluate the pharmacokinetics (PK) of the combination.

**Results:**

Dose-limiting toxicities were transaminitis and cutaneous toxicity. The most frequently reported adverse events were elevated transaminases, hypophosphatemia, fatigue, anorexia, diarrhoea, nausea, rash and palmar–plantar erythrodysaesthesia. Sirolimus did not change the PK of sorafenib; in contrast, sorafenib reduced the AUC_(0−96)_ and *C*_max_ of sirolimus. No objective responses were observed; eight patients showed stable disease for a median of 16.3 weeks (range 8–24). The MTD of the combination was sorafenib 200 mg b.i.d. with sirolimus 1 mg q.d.

**Conclusion:**

The combination of sorafenib and sirolimus showed enhanced toxicity, which could not be explained by the PK of both drugs. The relative low doses at the MTD, in combination with the PK results, do not warrant further development of this combination.

Multiple signalling pathways contribute to tumour growth and development. Single-agent strategies with small molecule signal transduction inhibitors or antibodies against one of these targets have shown clinical activity in several tumour types, although, only modest prolongation of overall survival has been shown. Combining agents that target pathways at multiple sites may enhance anti-tumour activity by biochemical and clinical synergism, reduce drug resistance or be successful with lower doses resulting in less toxicity (Dent *et al*, 2009). Sorafenib inhibits the receptor tyrosine kinases, vascular endothelial growth factor receptor 2 (VEGFR2), VEGFR3, Fms-related tyrosine kinase 3, c-KIT, platelet-derived growth factor receptor, and threonine kinases B-RAF and C-RAF. Sorafenib inactivates the RAS-RAF-MEK-ERK pathway, with subsequent inhibition of tumour-cell proliferation and angiogenesis. It has been approved for the second-line treatment in metastatic renal-cell cancer and hepatocellular carcinoma. The recommended dose of sorafenib is 400 mg twice daily (b.i.d.) in metastatic renal-cell cancer. Sirolimus is an orally administered mammalian target of rapamycin (mTOR) inhibitor and is the active metabolite of intravenously administered temsirolimus, registered for first-line treatment in poor prognosis metastatic renal-cell cancer. The threonine kinase mTOR is a key element of the intracellular signalling pathways involved in tumour cell proliferation, growth, survival and angiogenesis. Its activation leads to progression from the G_1_ to S phase of the cell cycle (Abraham and Gibbons, 2007). Mammalian target of rapamycin is activated aberrantly in tumours. This activation is increased by several signalling pathways, including the phosphatidyl-inositol 3-kinase/Akt, epidermal growth factor, Ras/mitogen-activated protein kinase pathways (Meric-Bernstam and Gonzalez-Angulo, 2009). One of the most important downstream proteins affected by mTOR is hypoxia-induced factor 1*α*. Hypoxia-induced factor 1*α* is a transcription factor, essential for the expression of genes necessary for cell growth in hypoxic conditions, as in tumours. Transcription of *VEGF* gene is regulated by hypoxia-induced factor 1*α* (Forsythe *et al*, 1996). Mammalian target of rapamycin is an attractive target for anti-cancer therapy. Sirolimus is approved as an immunosuppressive agent indicated for the prophylaxis of organ rejection in renal transplant patients. Currently sirolimus is studied in several types of cancer at doses of 0.5–10 mg orally daily (Stallone *et al*, 2005; Reardon *et al*, 2006; Rizell *et al*, 2008).

Combined inhibition of the VEGFR- and mTOR- signalling network may work synergistically. During treatment with a VEGFR inhibitor, such as sorafenib, plasma levels of VEGF will increase, which may contribute to resistance (Sosman and Puzanov, 2009). By addition of an mTOR inhibitor, the VEGF production will be downregulated by the effect of mTOR inhibition on hypoxia-induced factor 1*α*. On the basis of pre-clinical data and different mechanisms of anti-tumour activity of sorafenib and sirolimus, the current phase I trial was performed with this combination. Special attention was paid to the partly overlapping toxicity profiles and the fact that both drugs are metabolized by CYP3A4. The primary objective of this study was to identify the recommended dose of the combination of sorafenib and sirolimus for subsequent phase II studies. The secondary objectives were to (i) analyse the pharmacokinetic (PK) profiles, (ii) determine the safety profile (ii) determine the maximum tolerated dose (MTD) and (iv) evaluate preliminary activity of the combination of sorafenib and sirolimus.

## Patients and methods

### Patient eligibility

Patients aged ⩾18 years, with histologically or cytologically confirmed advanced solid malignancies refractory to conventional treatment or without any regular therapy option were enrolled. Eligibility criteria included life expectancy ⩾12 weeks, Eastern Cooperative Oncology Group performance status ⩽1, no previous anti-cancer therapy within 4 weeks of study entry, no previous treatment with sorafenib or sirolimus, adequate hematopoietic (absolute neutrophil count ⩾1.5 × 10^9^ l^−1^; platelets ⩾100 × 10^9^ l^−1^), hepatic (bilirubin ⩽1.5 × upper limit of normal (ULN), aspartate transaminase/alanine transaminase ⩽2.5 × ULN, in case of liver metastases ⩽5 × ULN,) and renal function (creatinine ⩽2 × ULN). Patients with clinically symptomatic brain tumours or brain metastases and patients with uncontrolled comorbidity were excluded. The local ethics committee approved the study protocol. Written informed consent was obtained from all patients before any study-related procedures.

### Study design and dose-escalation schedule

**Figure 1:**
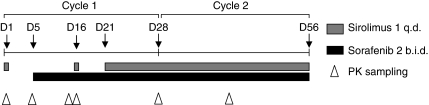
Treatment schedule and pharmacokinetic (PK) schedule. The DLT period ended after 28 days of combination of sorafenib and sirolimus administration, thus, 50 days after start of study treatment.

The recommended dose of the combination of sorafenib and sirolimus was determined by dose escalation. Patients were initially treated in cohorts of six per dose level. The number six was chosen to achieve adequate PK profiling of the combination of drugs, especially owing to the described large variances in sirolimus PK parameters (MacDonald *et al*, 2000). Both drugs were administered orally. Initially planned dose levels (DLs) were as follows: DL 1 (starting DL): sorafenib 200 mg b.i.d. and sirolimus 2 mg q.d., DL 2: sorafenib 400 mg b.i.d. and sirolimus 2 mg q.d., and DL 3: sorafenib 400 mg b.i.d. and sirolimus 4 mg q.d. In case DL 1 would not be feasible, a DL 0 was defined with sorafenib 200 mg b.i.d. and sirolimus 1 mg q.d. An extra intermediate DL (DL_im_) was amended: DL_im_: sorafenib 400 mg b.i.d. and sirolimus 1 mg q.d. During the first cycle, a run-in period was used for optimal PK analysis of both single drugs as well as to investigate the influence of sorafenib on sirolimus and vice versa (Figure 1). Therefore, in the first cycle, sirolimus was administered on day 1, once on day 16 and from day 21 as per a daily continuous schedule, while sorafenib was administered from day 5 on a daily continuous schedule. (Figure 1) The duration of one cycle was 28 days. The dose limiting toxicity (DLT) period ended after 28 days of administration of combination of sorafenib and sirolimus, thus 50 days after start of the study treatment. This prolonged DLT period was chosen because of the expectation that the first 7 days were needed to reach a steady concentration of sirolimus.

Patients remained in study medication for as long as the investigator felt it was in their best interest and there was no evidence of progressive disease (PD) or unacceptable toxicity.

### Definition of MTD and dose-limiting toxicity

Toxicity was evaluated according to the common toxicity criteria for adverse events (AEs) (CTCAEv3.0). The MTD was defined as the highest dose at which none or one out of six patients developed a DLT.

DLT was defined as any of the following events that was determined to be possibly or probably related to sorafenib and/or sirolimus and occurred during the first 50 days of treatment: any non-haematological grade 3–4 toxicity with the exceptions of nausea and vomiting, and fever that could be rapidly controlled with appropriate measures, grade 4 neutropenia lasting for ⩾7 days or febrile neutropenia defined as absolute neutrophil count ⩽1.0 × 10^9^ l^−1^ and fever ⩾38.5°C; grade 4 thrombocytopenia and grade 4 uncontrolled hypertension. The protocol was amended to define that hypophosphatemia grade 3 or 4 was not a DLT, as this is a well-known toxicity of sorafenib (Escudier *et al*, 2007).

### Patient evaluation and follow-up

Toxicity assessment, haematology and clinical biochemistry were performed at baseline and weekly during the first two cycles, and thereafter once every 2 weeks. Full physical examination and ECOG performance status were recorded at baseline and before each new cycle. Concomitant medications were recorded at every visit.

Response was evaluated according to the Response Evaluation Criteria in Solid Tumours (Therasse *et al*, 2000) at baseline and after every second cycle.

### Pharmacokinetic assessments

Sorafenib is metabolized primarily in the liver and undergoes oxidative metabolism, mediated by CYP3A4, as well as glucuronidation by UGT1A9. The elimination half-life is 25–48 h. Sirolimus is a substrate for both CYP3A4 and P-glycoprotein. It is extensively metabolized by *O*-demethylation and/or hydroxylation. The terminal half-life in stable renal transplant recipients was 62±12 h, however, the effective half-life is shorter and the mean steady-state concentrations are achieved after 5–7 days.

To evaluate the single-agent PK of sirolimus, blood samples were obtained at 15 time points for up to 4 days after the first administration, as follows: at baseline, 20 and 40 min, and at 1, 2, 3, 4, 6, 8, 12, 15, 24, 36, 48, 72 and 96 h after the first administration on day 1. On day 15, the PK of single-agent sorafenib was analysed by collecting blood samples at baseline and 30 min, at 1, 2, 4, 8, 10 and 12 h after administration of sorafenib. To evaluate continuous dosing of sorafenib combined with single-dose sirolimus, blood samples were collected starting on day 16, when patients were already treated with sorafenib since day 5 and single-dose sirolimus was added. Samples were obtained at same time points after day 1 for sirolimus, and at baseline after 30 min and at 1, 2, 4, 8, 12 and 24 h for sorafenib. Fully validated LC/MS/MS assay methods were applied to quantify blood concentrations of sirolimus and plasma concentrations of sorafenib. Lower limits of quantization were 0.26 *μ*g l^−1^ for sirolimus and 0.01 mg l^−1^ for sorafenib.

On days 1 and 15 of the second cycle, samples were collected to determine steady-state concentrations of sorafenib in plasma and of sirolimus in blood after combined continuous dosing at time points as follows: baseline, after 20, 30 and 40 min, and after 1, 2, 3, 4, 6, 8, 10 and 12 h after administration. In this way, we collected data of both drugs on PK of single-agent sirolimus, single-agent sorafenib, effect of single-dose sirolimus on steady-state level of sorafenib and effect of combination therapy on steady-state plasma or blood levels. Peak plasma or blood concentrations (*C*_max_), overall drug exposure (area under the plasma concentration *vs* time curve; AUC) and terminal plasma half-life (*t*_1/2_) were calculated using non-compartmental methods according to the Bayer guideline ‘Harmonization of Data Evaluation in Pharmacokinetics – A Task Force Report – ’ (1992 R-Report No. R-5747 and 2000 Amendment A to Report No. R-5747). A paired *t*-test on log-transformed values was used to calculate changes in AUC, *C*_max_ and *t*_1/2_. A *P*-value ⩽0.05 was considered significant.

## Results

### General trial conduct

Between July 2007 and May 2009, 20 patients were screened for inclusion in the trial. A total of 19 patients started treatment as one patient had a screening failure because of the appearance of clinically symptomatic brain metastases. Patient characteristics are summarised in Table 1. Three patients had early PD within the evaluation period of the first 50 days and were replaced as per protocol. One patient was not evaluated for DLT owing to incorrect medication intake by the patient herself. One patient (7%) received one cycle, nine patients (60%) received two cycles, three patients (20%) received four cycles and two patients (13%) received six cycles. No relation between number of cycles received and DLs was apparent.

### Dose-limiting toxicities and MTD

On DL 1 (sorafenib 200 mg b.i.d., sirolimus 2 mg q.d.) three out of six patients experienced a DLT. Two patients developed a grade 3 elevation of aspartate transaminase, and one patient a grade 3 elevation of alanine transaminase starting around day 28, that is 7 days after starting the combination treatment. In one patient this was accompanied by grade 3 fatigue, grade 3 anorexia and grade 3 weight loss. In another patient experiencing a DLT, grade 3 anorexia was also present. In all three patients, the transaminases values returned to baseline levels after discontinuation of sorafenib and sirolimus.

As this dose level was not tolerated, we decreased to DL 0 (sorafenib 200 mg b.i.d., sirolimus 1 mg q.d.). One out of six patients had a DLT due to cardiac ischaemia. This 49-year-old female patient with controlled hypertension and a chondrosarcoma presented on day 48 of the study, with chest pain due to cardiac ischaemia. A coronary angiography showed a small occlusion of the left coronary artery due to arterial thrombosis, and apical ballooning. The patient discontinued the study medication and recovered completely.

**Figure 2:**
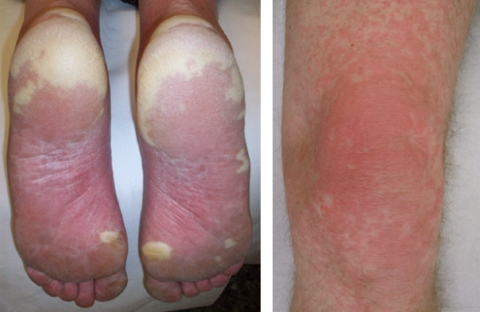
Left panel: Plantar palmar erythrodysaesthesia (PPE, also called ‘hand foot syndrome’). Right panel: Acneiform dermatitis.

As DL 0 was tolerated, we amended the protocol to escalate to an DL_im_ (sorafenib 400 mg b.i.d., sirolimus 1 mg q.d.), as the registered regular dosing of sorafenib is 400 mg b.i.d. On this DL_im_, three out of four patients experienced a DLT. All three patients experienced a grade 3 palmar–plantar erythrodysaesthesia (PPE, also called ‘hand–foot syndrome’ Figure 2), in one patient accompanied with grade 3 acneiform dermatitis (Figure 2). One of the patients also had a grade 3 fatigue. In all three patients, the PPE recovered completely after discontinuation of the study drugs. Consequently, the MTD was established as sorafenib 200 mg b.i.d. and sirolimus 1 mg q.d.

### Overall safety and tolerability

#### Adverse events and serious AEs.

All patients experienced several AEs. Table 2 summarizes AEs occurring with a frequency >30% or grade 3 or worse. The most frequently reported AEs were elevated aspartate transaminase and alanine transaminase (95% and 63%), anaemia (89%), hypophosphatemia (84%), anorexia (80%), cough (79%), fatigue (79%), PPE (69%), nausea (68%) and diarrhoea (68%).

As shown in Table 3, the frequency of anorexia, fatigue, diarrhoea and PPE was high in the first 50 days on study treatment, and increased significantly during prolonged study participation. Most patients had a combination of several grade 1–3 toxicities. Therefore, the combination therapy was intolerable for most patients.

#### Liver test abnormalities.

Abnormalities in hepatic function tests were frequently observed, especially on DL 1. As early as 1 week after the combined dosing, elevation of aspartate transaminase and alanine transaminase were observed with peak values between days 28–42 (2–4 weeks of combination therapy). Two of the three patients with grade 3 elevations of transaminases suffered from hepatocellular carcinoma and had baseline transaminase values between 1.0–2.5 × ULN. The third patient had colorectal cancer without liver involvement and with a normal hepatic function at baseline. In these three patients, hepatic function restored to baseline after cessation of therapy within weeks. Furthermore, recovery of hepatic function was also observed in patients with less enhanced elevations of transaminases, who did not interrupt the combination treatment, generally between 2–6 weeks after measurement of peak liver function abnormalities.

#### Cutaneous toxicity.

PPE, maculopapular rash, acne, pruritis and dry skin were the frequently reported AEs with combined sorafenib and sirolimus. In general, skin toxicity was mild. However, on the DL_im,_ severe PPE and rash occurred, generally starting within 10 days after initiation of the combination therapy. The patients experienced a painful red skin, scaling with yellowish demarcations (Figure 2). Emollients did provide some relief, although short drug interruptions of 3–5 days were needed for full clinical recovery. In two patients, sorafenib was reinitiated at a lower dose of 200 mg b.i.d., but one of them had a recurrence, after which we permanently discontinued the sorafenib in this patient.

### Pharmacokinetics

#### The effect of sirolimus on sorafenib PK.

On average, concomitant administration of single oral doses of 1 mg or 2 mg of sirolimus did not influence AUC_(0−12 h)_ (*P*=0.67) and *C*_max_ (*P*=0.96) of sorafenib following multiple dosing with 200 mg b.i.d. of sorafenib. In the DL_im_, when 1 mg sirolimus was administered together with 400 mg b.i.d. of sorafenib, minor non-significant mean decreases in both parameters by 15% (*P*=0.14) and 8% (*P*=0.57) were observed. (Table 3)

#### The effect of sorafenib on sirolimus PK.

On DL 1, pre-treatment with multiple oral doses of 200 mg b.i.d. sorafenib resulted in a mean decrease of *C*_max_ of sirolimus by 55% (*P*=0.006), while the AUC_(0−96 h)_ of sirolimus non-significantly decreased by 37% (*P*=0.125) and the mean terminal half-life of sirolimus remained unchanged (*P*=0.66). Similarly, on the DL_im,_ the *C*_max_ decreased on average by 55% (*P*=0.027) and the AUC_(0−96 h)_ by 25% (*P*=0.363). This is in contrast to results from patients on DL 0, in which no change in the mean AUC_(0−96 h)_ of sirolimus was observed (*P*=0.955) on simultaneous administration of both drugs, and *C*_max_ was only non-significantly reduced by 18% (*P*=0.515) on average (Table 4).

### Anti-tumor activity

In all, 14 patients were evaluated for response after two cycles. No objective responses were observed. Six patients had PD at the first evaluation after 8 weeks (two cycles). Of the eight remaining patients, two patients with stable disease decided to stop after 9 and 18 weeks of study participation because of the considerations on the quality of life based on multiple grade 1–2 toxicities. One other patient had to stop after 8 weeks because of cardiac ischaemia, which was a DLT in the last few days of the DLT period. Of the remaining four patients, two showed PD after 16 weeks (four cycles) and two after 24 weeks (six cycles).

## Discussion

This phase I study investigates the combination of two oral small molecule signal transduction inhibitors each targeting different pathways, namely the RAS-RAF-MEK-ERK and VEGF pathway (sorafenib) and the phosphatidyl-inositol 3-kinase/Akt pathway (sirolimus). In this study, the combination of sorafenib and sirolimus caused enhanced toxicity with only modest clinical activity.

The recommended dose was established on sorafenib 200 mg b.i.d. and sirolimus 1 mg q.d. This is 50% below the recommended dose for single-agent therapy with sorafenib. For sirolimus, as an anti-cancer therapy, no regular dosing is known, although reports mention effective doses between 0.5 and 10 mg daily (Stallone *et al*, 2005; Reardon *et al*, 2006; Rizell *et al*, 2008). The PK results did not provide a clarification for the observed enhanced toxicity.

The observed toxicity of the combination of sorafenib and sirolimus was impressive. This is illustrated by Table 2, showing high frequencies of toxicities, which accumulated during prolonged study participation. The impact of the ongoing combination of several toxicities, even when relatively mild as grade 1 or 2, on the quality of life of the participating patients was high. Enhanced toxicity is observed in several other combinations of targeted therapies. A recent phase I study, which combined sorafenib with monoclonal VEGF antibody bevacizumab reported enhanced toxicity, including transaminitis grade 2–4 in 13 out of 39 patients (33%) and PPE in 31 out of 39 patients (79%, in 23 patients grade ⩾2; Azad *et al*, 2008) Like in our study, the MTD of this combination was below single-agent therapy doses with sorafenib 200 mg b.i.d. and bevacizumab 5 mg kg^−1^ intravenously once in every 2 weeks. The same group studied intermittent dosing of sorafenib with bevacizumab, which was tolerated better (Lee *et al*, 2010). In two preliminary reports of studies combining sorafenib with mTOR inhibitor temsirolimus, significant toxicity was described including mucocutaneous toxicity, serum transaminase elevations, hypertrygliceridemia and thrombocytopenia (Patnaik *et al*, 2007; Kim *et al*, 2009). In the first study, the MTD was sorafenib 400 mg and 200 mg daily with temsirolimus 25 mg intravenously every week. (Kim *et al*, 2009) In the second study, the MTD was not yet determined, although the DLs combining sorafenib 400 mg b.i.d. with temsirolimus 25 mg intravenously every week were not tolerated and lower doses of sorafenib were under study (Patnaik *et al*, 2007). In contrast to these observations, three preliminary studies reported the combination of sorafenib and mTOR inhibitor everolimus as tolerable and safe, as did one preliminary report of a phase I study with the combination of sorafenib and temsirolimus (Giessinger *et al*, 2008; Cen *et al*, 2009; Harzstark *et al*, 2009; Wen *et al*, 2009). Two studies combined sunitinib with temsirolimus. In one of them, PPE and fatigue were common AEs. The other study was terminated because of substantial toxicity (Li *et al*, 2009; Patel *et al*, 2009).

We can only speculate about the reasons for the enhanced toxicity. Toxicities of targeted therapies might be because of the inhibition of multiple so-called ‘off-targets’ elements in the pathways that are not the primary target of a tyrosine kinase inhibitor. Both specific targets as well as off-targets do have a normal physiological function, for example the survival of endothelial cells and the maintenance of vascular integrity. The combination of several targeted agents enhances the number of elements within the pathways that are influenced, which might explain the enhanced toxicity (Gotink and Verheul, 2009). Furthermore, in a ‘vertical strategy’, drugs are combined to inhibit a cascade of signalling molecules. Here applied combination of mTOR inhibitor sirolimus and VEGFR inhibitor sorafenib is an example for this approach (Sosman and Puzanov, 2009). By inhibition in a ‘vertical’ strategy, feedback loops in the network of signalling pathways might be involved in unexpected and undesirable outcomes of targeted therapies (Gotink and Verheul, 2009). Another interesting factor involved in toxicity might be an altered pharmacodynamic effect of a tyrosine kinase inhibitor owing to polymorphisms in specific genes encoding for metabolizing enzymes, efflux transporters and drug targets, as is observed for sunitinib (van Erp *et al*, 2009). The combination of several targeted therapies with overlapping metabolic profiles may enhance gene vulnerability.

In this study, the most occurring dose-limiting toxicities were serum transaminase elevations and PPE. Both toxicities have been reported before in combination strategies with mTOR inhibitors and VEGF(R) inhibitors (Patnaik *et al*, 2007; Azad *et al*, 2008, 2009; Kim *et al*, 2009). However, for both sorafenib and sirolimus, serum transaminase elevations are not a frequently reported AE (Bhojani *et al*, 2008). The timing of the rise in serum transaminases 2–3 weeks after start of the combination and the recovery after discontinuation of the combination, suggest a causal relationship. Vascular endothelial growth factor has a role in structural, functional integrity of the liver. Most consistently, growth stimulating, regenerative and cytoprotective effects of VEGF and a VEGFR agonist have been found in pre-clinical models of ischaemia and reperfusion-induced hepatic toxicity, indicating a causal relationship between VEGFR inhibitors and transaminitis (Eskens and Verweij, 2006). Analysis of skin biopsies of PPE patients indicate that epidermal cells are swollen, capillaries are dilated and apoptotic endothelial cells are present. This suggests that the skin toxicity is a direct consequence of the biological activity of VEGFR inhibitors (Faivre *et al*, 2006). Other observed cutaneous toxicities were maculopapular rash, acne, pruritis and dry skin. These are well known AEs of oral VEGFR inhibitors. (Lee *et al*, 2010).

The clinical efficacy observed in our study was only moderate, with stable disease in 53% of patients but only in 14% persisting for more than 4 months. The patients participating in this study were heavily pre-treated and had PD at the time of enrolment.

In conclusion, the MTD of the combination is sorafenib 200 mg b.i.d. and sirolimus 1 mg q.d. This combination showed enhanced toxicity, which could not be explained by the influence of the PK of each targeted agent. We do not recommend further exploration of this dosing schedule. Pre-clinical research focusing on the causes of enhanced toxicity of these combination therapies is highly warranted.

## Figures and Tables

**Table 1:** Patient characteristics (*n*=19)

Age, median (range)	49 (28–64)
Male/female	11/8
	
*Performance score*	
0	14 (74%)
1	5 (26%)
	
*Tumour type*	
Sarcoma	8 (42%)
Colorectal cancer	3 (16%)
Melanoma	2 (11%)
Non-small cell lung cancer	2 (11%)
Hepatocellular carcinoma	2 (11%)
Thyroid cancer	1 (5%)
Breast cancer	1 (5%)
	
Previous treatment	19 (100%)
*Systemic treatment*	
Chemotherapy	15 (79%)
Targeted therapy	3 (16%)
Hormonal treatment	2 (11%)
Surgery	17 (89%)
Radiotherapy	9 (47%)

**Table 2:** Haematological and non-haematological adverse events occurring in >30% of patients or reaching grade 3–4 severity. (*n*=19)

	**DLT period (first 50 days)**					**All cycles**				
**Adverse event**	**Grade 1 (*n*)**	**Grade 2 (*n*)**	**Grade 3 (*n*)**	**Grade 4 (i)**	**All (%)**	**Grade 1 (*n*)**	**Grade 2 (*n*)**	**Grade 3 (*n*)**	**Grade 4 (*n*)**	**All (%)**
*Haematological adverse events*										
Anaemia	9	4	0	0	68	9	8	0	0	89
Neutropenia	0	2	0	0	11	4	2	0	0	32
										
*Non-haematological adverse events*										
Anorexia	7	0	2	0	47	7	6	2	0	79
Nausea	7	3	0	0	53	8	5	0	0	68
Vomiting	2	1	1	0	21	7	1	1	0	47
Dysphagia	4	0	0	0	21	6	0	1	0	37
Diarrhoea	5	4	0	0	47	7	5	1	0	68
Constipation	3	0	0	0	16	6	0	0	0	32
Weight loss	5	0	1	0	32	7	1	1	0	47
Fatigue	2	6	2	0	53	2	8	5	0	79
Acne	4	1	1	0	32	2	1	1	0	32
Dry skin	6	1	0	0	37	8	1	0	0	47
Rash	4	2	0	0	32	6	4	0	0	53
PPE	6	2	3	0	58	6	2	5	0	68
Alopecia	3	1	0	0	21	9	1	0	0	53
Hoarseness	5	1	0	0	32	6	1	0	0	37
Cough	11	1	0	0	63	14	1	0	0	79
Dyspnoea	7	0	0	0	37	8	0	0	0	42
Abdominal pain	5	4	0	0	47	6	6	0	0	63
Thoracic pain	1	0	0	0	5	7	1	0	0	42
Dizziness	5	0	0	0	26	6	0	0	0	32
Hypertension	1	1	4	0	32	1	1	5	0	37
Cardiac ischaemia	0	0	0	1	5	0	0	0	1	5
Sensory neuropathy	6	0	0	0	32	7	1	0	0	42
Elevated AST	10	2	3	0	79	12	3	3	0	95
Elevated ALT	9	1	1	0	58	10	1	1	0	63
Elevated bilirubin	2	3	0	0	26	3	3	0	0	32
Hypoalbumin	3	5	0	0	42	4	6	1	0	58
Hypophosphatemia	0	11	4	0	79	0	12	4	0	84
Hypokalemia	7	0	0	0	37	9	0	0	0	47
Hypercholesterolaemia	7	1	0	0	42	7	2	0	0	47
Hypertryglyceremia	4	2	0	0	32	6	2	0	1	47

Abbreviations: ALT, alanine transaminase; AST, aspartate transaminase.

**Table 3:** Pharmacokinetics of sorafenib, expressed as geometric means with associated 95% CI

		**AUC_(0–12 h)_ mg h l^−1^ (95% CI)**					**C_max_ mg l^−1^ (95%CI)**				
		**Cycle 1**			**Cycle 2**		**Cycle 1**			**Cycle 2**	
**DL**	**Dose**	**Day 15**	**Day 16**	**Ratio d15/d16**	**Day 1**	**Day 15**	**Day 15**	**Day 16**	**Ratio d15/d16**	**Day 1**	**Day 15**
1	Sor 200 mg b.i.d., sir 2 mg q.d	47.1 (33–69) (*n*=6)	47.9 (31–79) (*n*=6)	1.02 (0.81–1.26)	39.3 (21–74) (*n*=4)	66.9 (66.3–67.5) (*n*=2)	6.04 (4.1–8.9) (*n*=6)	6.00 (4.09–8.8) (*n*=6)	1.007 (0.76–1.31)	5.15 (2.9–9.2) (*n*=4)	7.27 (6.5–8.1) (*n*=2)
0	Sor 200 mg b.i.d., sir 1 mg q.d.	63.8 (45–89) (*n*=6)	64.9 (55–77) (*n*=6)	1.02 (0.82–1.25)	58.4 (50–68) (*n*=6)	56.7 (42–76) (*n*=6)	7.59 (5.0–11.5) (*n*=6)	7.98 (6.4–10.0) (*n*=6)	1.05 (0.82–1.35)	7.12 (5.7–8.8) (*n*=6)	7.32 (4.9–10.9) (*n*=6)
IM	Sor 400 mg b.i.d., sir 1 mg q.d.	102 (59–178) (*n*=4)	86.6 (48–155) (*n*=4)	0.85 (0.72–0.99)	74.9 (36–157) (*n*=4)	48.4 (47–50) (*n*=2)	13.0 (7–23) (*n*=4)	11.9 (7–20) (*n*=4)	0.92 (0.7–1.2)	6.64 (5.0–18.7) (*n*=4)	5.55 (5.2–5.9) (*n*=2)

Abbreviations: CI=confidence interval; IM=intermediate; sir=sirolimus; sor=sorafenib.

Cycle 1, day 15: continuous dosing sor, day 16: effect of single-dose sir on continuous dosing sor; cycle 2, days 1 and 15: effect of continuous dosing sir on continuous dosing sor.

**Table 4:** Pharmacokinetics of sirolimus expressed as geometric means with associated 95% CI

		**AUC *μ*g h l^−1^ (95%CI)**					***C*_max_ *μ*g l^−1^ (95%CI)**					**t ½ h (95%CI)**	
		**AUC_(0–96 h)_**			**AUC_(0–12 h)_**								
		**Cycle 1**			**Cycle 2**		**Cycle 1**			**Cycle 2**		**Cycle 1**	
**DL**	**Dose**	**Day 1**	**Day 16**	**Ratio d1/d16**	**Day 1**	**Day 15**	**Day 1**	**Day 16**	**Ratio d1/d16**	**Day 1**	**Day 15**	**Day 1**	**Day 16**
1	Sor 200 mg b.i.d., sir 2 mg q.d.	124 (102–151) (*n*=6)	78.2 (55–111) (*n*=6)	0.63 (0.39–1.03)	88.2 (50–155) (*n*=4)	84.6 (*n*=1)	5.74 (4.8–6.8) (*n*=6)	2.60 (1.9–3.6) (*n*=6)	0.45 (0.33–0.63)	10.0 (6.4–15.7) (*n*=5)	17.6 (*n*=2)	50.5 (43.7–58) (*n*=6)	52.5 (42.9–64.3) (*n*=6)
0	sor 200 mg b.i.d., sir 1 mg q.d.	91.1 (59–141) (*n*=6)	91.7 (67–125) (*n*=6)	1.01 (0.81–1.26)	59.4 (47–75) (*n*=6)	57.8 (41–81) (*n*=6)	3.81 (2.6–5.6) (*n*=6)	3.14 (2.4–4.2) (*n*=6)	0.82 (0.48–1.42)	6.30 (4.8–8.2) (*n*=6)	6.36 (4.7–8.5) (*n*=6)	45.5 (27–75) (*n*=6)	56.2 (39–82) (*n*=6)
IM	sor 400 mg b.i.d., sir 1 mg q.d.	139 (98–239) (*n*=3)	104 (100–113) (*n*=3)	0.75 (0.46–1.14)	101 (55–115) (*n*=2)	113 (*n*=2)	7.99 (6.0–13.6) (*n*=3)	3.62 (2.4–4.3) (*n*=3)	0.45 (0.41–0.58)	11.2 (5.7–13.0) (*n*=2)	13.7 (10.5–18.0) (*n*=2)	38.30 (29.9–50.6) (*n*=3)	32.9 (24.5–52.7) (*n*=3)

Abbreviations: CI=confidence interval; IM=intermediate; sir=sirolimus; sor=sorafenib.

Cycle 1, day 1: single-dose sir, day 16: effect of continuous dosing sor on single-dose sir; cycle 2, days 1 and 16: effect of continuous dosing sor on continuous dosing sir.
